# Effects of codon optimization on coagulation factor IX translation and structure: Implications for protein and gene therapies

**DOI:** 10.1038/s41598-019-51984-2

**Published:** 2019-10-29

**Authors:** Aikaterini Alexaki, Gaya K. Hettiarachchi, John C. Athey, Upendra K. Katneni, Vijaya Simhadri, Nobuko Hamasaki-Katagiri, Puja Nanavaty, Brian Lin, Kazuyo Takeda, Darón Freedberg, Dougald Monroe, Joseph R. McGill, Robert Peters, Jacob M. Kames, David D. Holcomb, Ryan C. Hunt, Zuben E. Sauna, Amy Gelinas, Nebojsa Janjic, Michael DiCuccio, Haim Bar, Anton A. Komar, Chava Kimchi-Sarfaty

**Affiliations:** 1 Center for Biologics Evaluation and Research, Food and Drug Administration, Silver Spring, MD USA; 2 Center for Gene Regulation in Health and Disease, Cleveland State University, Cleveland, OH USA; 3 University of North Carolina at Chapel hill, Chapel hill, NC USA; 4 Bioverativ, Cambridge, MA USA; 5 SomaLogic, Inc, Boulder, CO USA; 6 National Center of Biotechnology Information, National Institutes of Health, Bethesda, MD USA; 7 Department of Statistics, University of Connecticut, Storrs, CT USA

**Keywords:** Recombinant protein therapy, Gene expression

## Abstract

Synonymous codons occur with different frequencies in different organisms, a phenomenon termed codon usage bias. Codon optimization, a common term for a variety of approaches used widely by the biopharmaceutical industry, involves synonymous substitutions to increase protein expression. It had long been presumed that synonymous variants, which, by definition, do not alter the primary amino acid sequence, have no effect on protein structure and function. However, a critical mass of reports suggests that synonymous codon variations may impact protein conformation. To investigate the impact of synonymous codons usage on protein expression and function, we designed an optimized coagulation factor IX (FIX) variant and used multiple methods to compare its properties to the wild-type FIX upon expression in HEK293T cells. We found that the two variants differ in their conformation, even when controlling for the difference in expression levels. Using ribosome profiling, we identified robust changes in the translational kinetics of the two variants and were able to identify a region in the gene that may have a role in altering the conformation of the protein. Our data have direct implications for codon optimization strategies, for production of recombinant proteins and gene therapies.

## Introduction

The genetic code is redundant, with most amino acids being encoded by more than one (synonymous) codon, some by as many as six. There is considerable bias in the use of synonymous codons that varies among species, with some codons being used more frequently than others^1^. Rare codons are generally decoded by low abundance tRNAs^2–5^. The concentration of cognate tRNAs is thought to be one of the major determinants influencing the speed of translation^6,7^, and the decoding process for individual codons may also be modulated by factors that impact the thermodynamics of codon-anticodon interaction, such as GC content, wobble base pairing and tRNA modifications^8,9^. It appears, however, that codon pair usage may be another important determinant of elongation speed^10,11^, and mRNA secondary structure, particularly hairpin loops, may also affect elongation^12,13^.

Codon optimization, a common term for a set of recombinant DNA techniques in which multiple codons within a gene sequence are replaced by synonymous ones, aims to increase the rate and efficiency of protein translation by using more abundant codons^14,15^. This approach is commonly used by the biopharmaceutical industry to improve the cost efficiency of recombinant protein production. It had been generally considered to be inconsequential to the structure and function of the generated protein, due to the commonly accepted Anfinsen’s dogma that postulates that amino acid sequence alone is sufficient to determine protein structure^16^.

Paradoxically, the widespread adoption of codon-optimization occurred at the same time that an overwhelming amount of research demonstrated that synonymous mutations can and do affect protein function^17–19^. Multiple examples have been reported where synonymous codon substitutions cause disease^20–23^. A range of mechanisms, including alternative splicing^24^, mRNA-protein binding^25^, miRNA binding^26^, mRNA stability^25^, and translation efficiency^27^, may link synonymous variants to altered protein expression^28^. Nevertheless, in some cases synonymous changes may have effects beyond the level of expression^18,19,23^. There have been multiple reports in which the synonymous variation-induced pathology is driven by changes in translational kinetics, ultimately leading to altered protein conformation^20–23^. A diverse set of biophysical and biochemical techniques can be used to study the effects of synonymous mutations on protein expression, folding, and function. However, until recently there were no sufficiently precise methods to study how changes in codon usage affect translational kinetics. Ribosome profiling^29^ allows the study of translation kinetics at single-codon resolution, opening a window of opportunity for identifying regions on the gene where synonymous substitutions are most likely to alter protein conformation. It should be noted, however, that early ribosome profiling experiments did not reveal a clear correlation between ribosomal stalling and rare codons^30^. Improvements in the method, such as removing the translation arresting agent cycloheximide, revealed distinct stages in the translation process and provided more detailed resolution of the process^31^. Nevertheless, a correlation between ribosome pause sites and rare codons has been difficult to show, especially in mammalian cells^32,33^. Interestingly, it was reported that a codon can exhibit up to 26-fold variability in its translation rate depending upon its context within a transcript^34^, clearly highlighting the difficulty in assigning a representative decoding rate on each codon. Following recent improvements in the ribosome profiling method^35^, a deep-learning based approach was successful in predicting ribosome stalling and correlating it with codon usage, as well as with tRNA adaptation, codon co-occurrence, proline codons, mRNAN^6^-methyladenosine modification, RNA-binding proteins and protein secondary structure, further pointing to the complexity of the association^36^. It should be noted, however, that in yeast and bacterial systems, a correlation between ribosome pausing and codon rarity has been much easier to establish^33,37^.

In the present study, we used human blood coagulation factor IX (FIX, when referring to the protein and *F9* when referring to the gene), as a model to study the effects of codon optimization on the kinetics of protein translation and protein conformation. We chose FIX, because of its importance as a therapeutic protein. There are currently several marketed recombinant FIX drugs^38^ and additionally there are FIX gene therapies under clinical trials^39^.

By modifying the codon usage of *F9* (using a commercially available algorithm), codon pair usage, GC content and the nucleotide sequence of the gene drastically changed, resulting also in altered mRNA thermodynamic stability. We further observed that this codon optimization results in increased protein levels, upon expression in HEK293T cells, in comparison with the wild-type variant, and notably that the two proteins differ with respect to their conformation. Lastly, we employed ribosome profiling to examine the association between changes in translational kinetics to potential locations within the codon optimized gene that may be responsible for the observed conformational changes. The improved understanding of the effect of codon optimization on protein conformation that we have gained from this study may contribute to the development of safer and more efficient FIX therapeutics.

## Results

### Codon optimization of *F9* leads to a series of changes in gene characteristics

**Figure 1:**
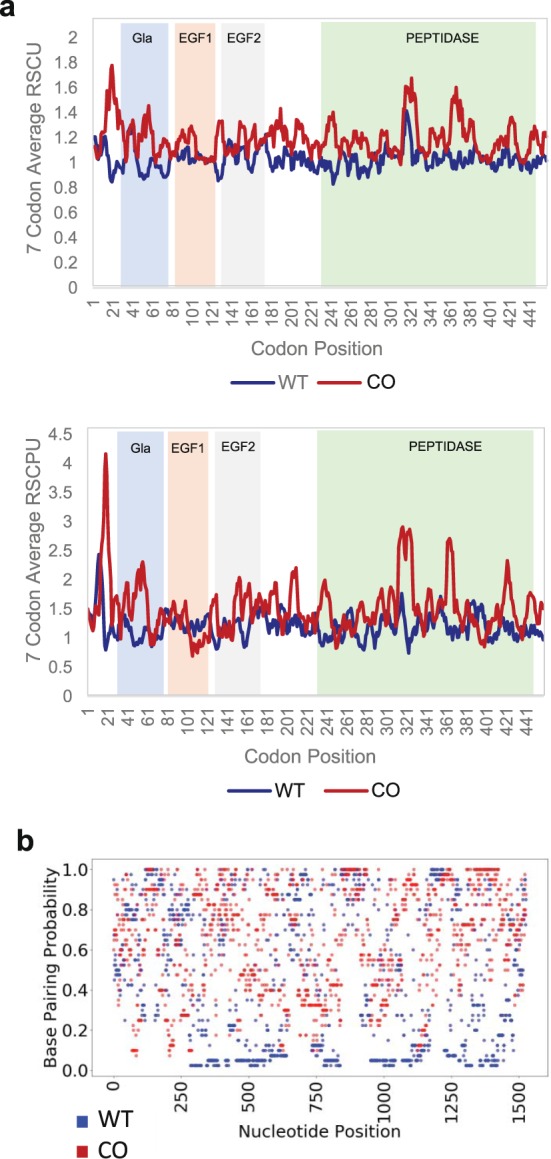
Properties of wild-type and codon-optimized *F9* genes’ sequences. (**a**) CO *F9* utilizes more common codons and common codon pairs. The Relative Synonymous Codon Usage (RSCU) and Relative Synonymous Codon Pair Usage (RSCPU) were calculated based on codon usage frequencies obtained from https://hive.biochemistry.gwu.edu/review/codon. The 7 codon and codon pair average of RSCU and RSCPU values were plotted for the WT and CO sequences of *F9*. (**b**) *In silico* analyses of mRNA equilibrium base-pairing probabilities were calculated based on RNAfold webserver (http://rna.tbi.univie.ac.at/cgi-bin/RNAWebSuite/RNAfold.cgi).

To study the effects of codon optimization on protein translation and conformation, we modified the human wild-type (WT) *F9* coding sequence (CDS) using a publicly available gene optimization algorithm (GeneArt/Fisher). This multiparametric optimization algorithm considers codon frequency, GC-content, avoiding UpA- and introducing CpG-dinucleotides, cryptic splice-sites, intragenic poly(A)-sites, direct repeats, RNA secondary structures and destabilizing elements, and internal ribosomal entry sites^40^. Similar to other publicly available tools^41,42^, the general aim is to enhance expression by increasing the translational rate and inhibiting mRNA degradation. The optimized sequence differed from the original sequence by 22.5% on the nucleotide level and by 60.9% on the codon level (Supplemental Fig. S1). In general, codon optimization leads to the omission of rare codons and enrichment of common ones. As a result, it leads to an increase in indices of codon usage. In this case, the Codon Adaptation Index (CAI) of the optimized sequence increased from 0.74 in the WT *F9* to 0.88 in the Codon Optimized (CO) *F9*. Similarly, the relative synonymous codon usage^43^ (RSCU) and the relative synonymous codon pair usage^44^ (RSCPU) metrics increased. Figure 1a illustrates the changes in RSCU and RSCPU along the *F9* gene, in relation to the location of the structural domains of the protein, gamma-carboxyglutamic acid (Gla), epidermal growth factor like-1 and 2 (EGF1 and 2) and peptidase. Interaction of FIX with Ca^2+^ occurs at its Gla domain, consisting of 12 modified Gla residues. The C-terminal half of the protein contains its catalytic domain which is a serine protease. Codon optimization also led to an increase in GC content, from 41.3% to 51.2%, and a decrease in the mRNA/open reading frame (ORF) minimum free energy (MFE), from −339.9 to −410.5 kcal/mol, (Supplemental Fig. 2) suggesting a more stable conformation. To further investigate the changes introduced by codon optimization in the mRNA structure, we calculated and plotted the equilibrium base-pairing probabilities of the WT and CO *F9* mRNAs (Fig. 1b). These metrics appeared to be significantly different between the two constructs (Wilcoxon signed-rank test p-value < 2.2e-16).

### Codon optimization of *F9* leads to enhanced expression in HEK293T cells

**Figure 2:**
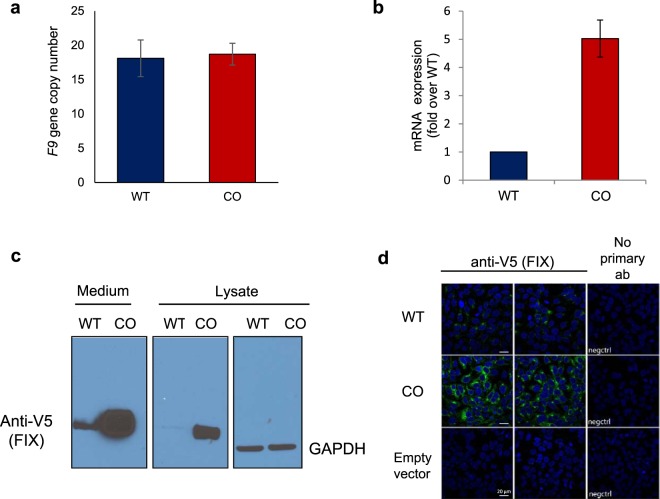
Factor IX high expression in HEK293T cells stably transfected with wild-type vs. codon-optimized *F9*. (**a**) Cell DNA was isolated and plasmid DNA copy numbers were determined by qPCR. The average of 3 experiments was plotted, s.e.m. are shown. (**b**) mRNA was isolated and analyzed by qPCR. The average of 3 replicates was plotted, s.e.m. are shown. (**c**) Cell supernatant (concentrated medium) and lysate was collected and analyzed by immunoblotting. Membranes were stained with anti-V5 or anti-GAPDH. (**d**) Cells were stained with anti-V5 primary antibody and Alexa488 conjugated donkey anti-mouse IgG (green) and examined by confocal microscopy. HEK293T cells stably transfected with empty vector were also included as a control.

We generated stably transfected HEK293T cells that expressed the WT and CO FIX. The WT and CO FIX expressing cell lines have on average similar integrated plasmid copy numbers (Fig. 2a). Three lines of evidence show that CO FIX was expressed at higher levels than WT FIX in these cells (Fig. 2): (i) CO *F9* mRNA levels were 5-fold higher compared to WT *F9* (Fig. 2b); (ii) An immunoblot (Fig. 2c and Supplemental Fig. S3) showed amplification of this difference on protein levels, both for the intracellular and secreted protein; iii) Immunostaining of fixed cells (Fig. 2d) similarly documented the increase of intracellular FIX in cells expressing the CO variant.

### Codon-optimized and wild-type FIX variants have conformational differences

**Figure 3:**
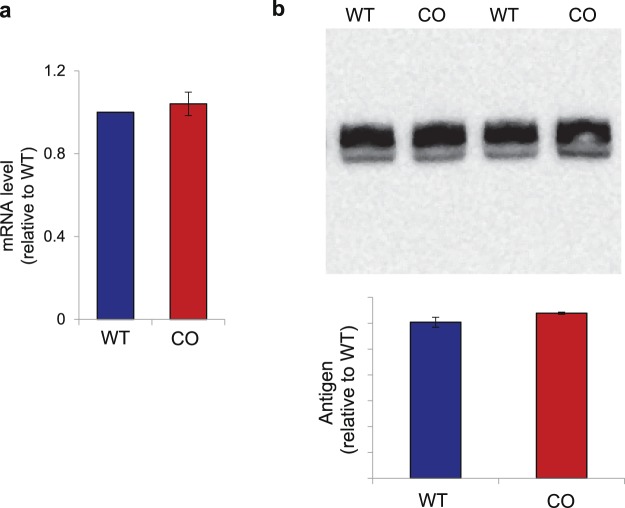
Generation of HEK293T cell clones expressing similar levels of wild-type and codon-optimized factor IX: (**a**) mRNA was isolated and analyzed by qPCR. The average of 3 replicates was plotted, s.e.m. are shown. (**b**) cell supernatant was collected and analyzed by immunoblotting. Membranes were stained with anti-V5 antibody. The average band intensity was plotted, s.e.m. are shown.

To assess the conformation of purified WT and CO FIX variant proteins we produced HEK293T cells that stably express similar levels of the WT and CO FIX variants. This precaution is necessary because differences in protein expression levels can lead to the saturation of the quality control machinery in the endoplasmic reticulum (ER)^45^, resulting is misfolded sub-populations of the overexpressed variant, hindering our confidence to attribute conformational changes to translational kinetics. To control for expression levels, HEK293T cells were transduced with a lentivirus carrying either the WT or the CO *F9* variants. Clones were expanded and screened for the levels of expression of the two variants. WT FIX and CO FIX clones with similar expression levels were further characterized. Relative transcript levels determined using qPCR confirmed that there were no detectable differences between WT and CO *F9* mRNA levels (Fig. 3a) in the two cell lines. Protein levels were also comparable (Fig. 3b).

We utilized the WT and CO FIX cell lines to purify FIX through its V5 tag at the 5′ which has been shown not to affect the molecule properties^46^. The use of linear V5 epitope allowed purification independently of possible conformational or functional differences between the two FIX variants.

**Figure 4:**
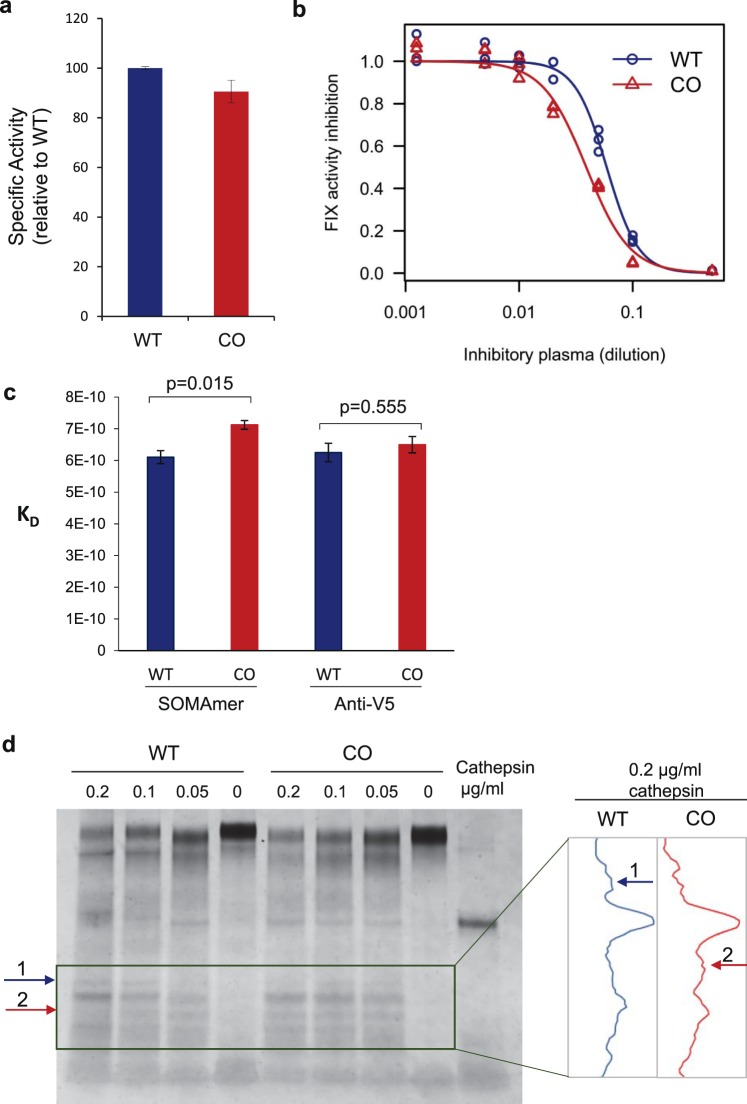
Codon-optimization leads to altered characteristics of factor IX protein. (**a**) WT and CO FIX activity were assessed with chromogenic assay. The average of 3 replicates was plotted, s.e.m. are shown. (**b**) WT and CO FIX were incubated with increasing amounts of plasma containing inhibitory antibodies, resulting in a dose dependent reduction of activity. Results from one (of three) representative experiment are shown. The EC_50_ was significantly different between WT and CO (WT EC_50_ = 0.059, CO EC_50_ = 0.039, p = 3.9e-09). (**c**) Binding affinities (KD) of WT and CO FIXs to the FIX-specific SOMAmer were significantly (p = 0.015) different. The anti-V5 antibody showed comparable (p = 0.555) affinities for the WT and CO FIX variants. (**d**) WT and CO FIX were exposed to the indicated concentrations of cathepsin. Samples were electrophoresed and silver stained to assess their pattern of digestion. The box highlights the area where most of the changes are observed. Band density was quantitated with ImageJ software. Arrow (1) points to a band that is present in the WT (cathepsin 0.2 μg/ml) lane and absent from the corresponding CO lane. Arrow (2) points to a band that is absent from the WT (cathepsin 0.2 μg/ml) lane and present the corresponding CO lane.

We found that WT and CO FIX exhibited comparable levels of specific activity (Fig. 4a). However, the two variants showed subtle but important differences in conformation, assessed with a range of bioanalytical techniques, such as antibody-mediated inhibition of FIX activity, aptamer binding and limiter proteolysis.

Since the development of anti-FIX antibodies significantly affects the safety of the therapeutic protein in hemophilia B patients^47^, we obtained FIX deficient plasma supplemented with anti-FIX inhibitory antibodies and compared the kinetics of interaction with WT and CO FIX. As expected, incubation of either variant with the inhibitory plasma, led to a decrease in activity (Fig. 4b). Interestingly, the kinetics of anti-FIX antibody-mediated inhibition were different for the two FIX variants (IC_50_ of 0.059 and 0.039for WT and CO FIX, p-value = 3.92E-09) (Fig. 4b), indicating that the two protein variants bind to inhibitory antibodies with different affinities, most likely due to conformational differences.

In recent years synthetic nucleic acid reagents called aptamers have emerged as surrogates to antibodies and appear to be particularly suited for bio-analytical applications. For instance, we and others have demonstrated that aptamers can be used to detect subtle differences in protein conformation^48–50^. Here we used next generation aptamers called SOMAmer^®^ (Slow Off-rate Modified Aptamer) reagents^51^ that target FIX, to compare the WT and CO FIX variants. Using BioLayer Interferometry to measure kinetic parameters of SOMAmer-FIX interactions, we observed a significant (p = 0.015) difference in the affinity of an anti-FIX-SOMAmer to purified WT and CO FIX variants (Fig. 4c). Conversely, no significant (p = 0.555) differences in affinities were detected when we measured the affinity of an anti-V5 antibody to the tag attached to both variants. These data also strongly suggest conformational differences between WT and CO FIX.

Another method that has been used to detect conformational differences in variants of the same protein is limited proteolysis^23^. This method relies on sites for proteolysis being either more exposed or buried because of alternate folding. Limited proteolysis of the purified WT and CO FIX with cathepsin revealed differential digestion patterns, detected by silver staining (Fig. 4d). Specifically, when the WT and CO variant were digested with a high concentration of cathepsin L, there was at least one fragment (Fig. 4d, arrow 1) that was unique to the WT variant and a smaller one (Fig. 4d, arrow 2) that was unique to the CO variant. The intensity of the bands was quantitated and plotted (Fig. 4d, right), clearly revealing the differences.

### Codon-optimized and wild-type FIX variants are translated with different kinetics

**Figure 5:**
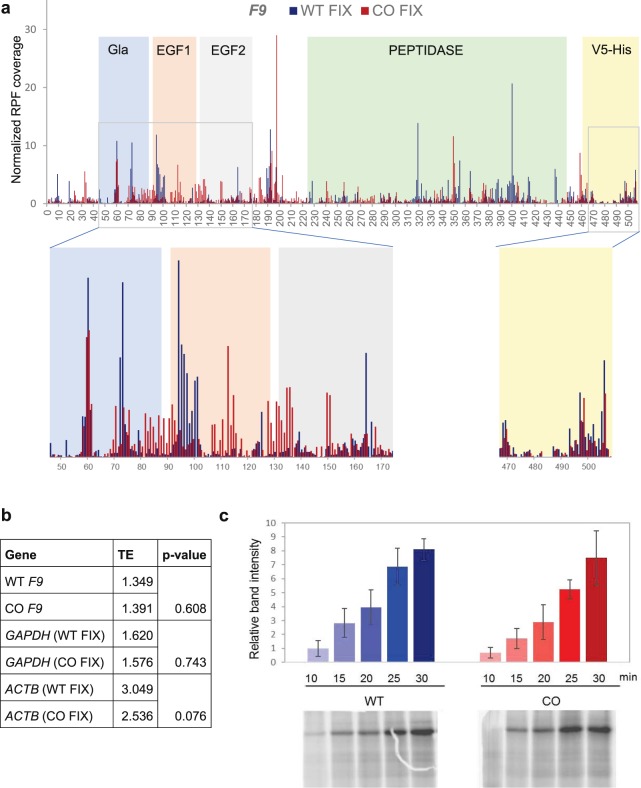
Ribosome profiling of wild-type and codon-optimized *F9* demonstrates drastically altered patterns of translation kinetics, but similar translation efficiency, in the two variants. (**a**) *F9* ribosome profiling data, shaded areas indicate the major FIX protein domains, Gla: γ-carboxyglutamic acid-rich, EGF: epidermal growth factor-like. Inserts show magnifications of the Gla-EGF1-EGF2 and His-V5 domains. (**b**) Translation efficiency (TE) of *F9*, *GAPDH* and *ACTB* in the WT and CO FIX expressing cell lines was calculated based on transcript mRNA and RPF abundance of thee independent experiments. (**c**) Representative [^35^S]-autoradiogram of the WT and CO FIX *in vitro* translation products in rabbit reticulocyte lysate (RRL) system (bottom panel) and quantitation analysis of the band intensities of three independent experiments (top panel). The average of 3 experiments was plotted, s.e.m. are shown.

Data presented above strongly indicate that the WT and CO FIX have different conformations. To elucidate what may be driving these differences, we compared the translational kinetics of the two variants using ribosome profiling. Ribosome profiling provides a snapshot of the distribution of ribosomes on mRNA at the codon level, from which translational kinetics can be extrapolated. Codon optimization of the *F9* gene led to an altered ribosomal distribution pattern from that of the WT transcript (Fig. 5a and Supplemental Fig. S4), suggesting significant changes in local translational kinetics. Conversely, unmodified housekeeping genes, *ACTB* and *GAPDH*, (in cell lines expressing WT and CO *F9* variants) exhibited comparable ribosome distribution profiles (Supplemental Figs S5 and S6). As an internal control the V5-His tag, which is found at the 5′ of the *F9* gene and has identical nucleotide sequence/codon composition in the two *F9* variants, also showed very similar ribosome distribution patterns (Fig. 5a, yellow shaded section).

Although we observed changes in local translational kinetics, the ribosome profiling data showed overall comparable transcript and ribosome protected fragment (RPF) occupancy, which indicates similar translational efficiencies (TE) between WT and CO *F9* (Fig. 5b and Supplemental Fig. S7a). This agrees with the *in vitro* translation studies (Fig. 5c), which demonstrate that the overall rates of translation between WT and CO FIX are similar. To confirm the quality of our ribosome profiling data, we also plotted the fragment length distributions for coagulation factor IX and for the whole genome Supplemental Fig. S7b,c). We further plotted the UTR and CDS distribution of the RPFs, which confirmed that most of the RPFs were within CDS (Supplemental Fig. S7d). In addition, the in-frame distribution of RPFs (Supplemental Fig. S7e) also supports the high quality of the data. Very tight correlation between two representative experiments, both for ribosome protected fragments (RPFs) and total mRNA, supports the reproducibility of the results (Supplemental Fig. S8).

We next investigated whether an association could be identified between ribosome pausing, in our gene of interest, and factors thought to be influencing translational kinetics. We considered RSCU as a measure of codon bias, RSCPU as a measure of codon pair bias, cognate tRNA abundance, mRNA equilibrium base-pairing probability and MFE as indicators of mRNA structure and stability. In addition, because we often noticed clustering of RPF coverage in our ribosome profiling plots, we considered whether RPF coverage for a certain codon would correlate with the coverage of preceding codons. We used these parameters as predictors and ran a step-wise model selection using the normalized RPFs as a response. RPF coverage in the two preceding codons was very significant in predicting the coverage of the following codon suggesting that there is clustering of codons of similar decoding time. Across the *F9* gene, RPF coverage did not significantly correlate with RSCU, RSCPU, equilibrium base-pairing probability or corresponding tRNA levels. Similar results were obtained with the control genes (Table 1 and Supplemental Table S1). A correlation between MFE values and ribosome profiling data was seen for WT *F9* gene but not for the control genes, *GAPDH* and *ACTB*. Taken together, these data highlight the complexity of predicting translational kinetics from nucleotide/codon sequence features.

**Table 1:** In a linear stepwise selection model, the ribosome occupancy of adjacent codons is the only consistently significant predictor of ribosome occupancy of a codon.

	Estimate	Std. Error	t-value	Pr( > |t|)	Significance
**WT** ***F9***					
(Intercept)	−0.89325	0.38834	−2.3	0.0218	*
RSCPU_Genome	−0.50998	0.27813	−1.834	0.0673	.
RSCPU_HEK_ERX	0.40442	0.27254	1.484	0.1385	
tRNA	−4.77239	2.88107	−1.656	0.0983	.
MFE_151	−0.02178	0.01039	−2.096	0.0366	*
Lag3	0.06721	0.04018	1.673	0.095	.
Lag1	0.48761	0.04027	12.109	<2e-16	***
**CO** ***F9***					
(Intercept)	−0.29018	0.05481	−5.295	1.78E-07	***
Lag2	0.16889	0.04401	3.838	0.00014	***
Lag1	0.39237	0.0439	8.939	<2e-16	***
***ACTB***					
(Intercept)	−0.99862	0.23398	−4.268	2.51E-05	***
RSCPU_HEK_ERX	0.10396	0.06776	1.534	0.12586	
Pair-prob	0.66064	0.25935	2.547	0.01126	*
Lag3	0.12719	0.04649	2.736	0.00652	**
Lag1	0.46591	0.04713	9.887	<2e-16	***
***GAPDH***					
(Intercept)	−0.55555	0.30251	−1.836	0.067199	.
RSCU_Genome	1.39465	0.61119	2.282	0.023139	*
RSCU_HEK_SRR	−1.33592	0.73108	−1.827	0.068558	.
RSCPU_Genome	−0.66171	0.27595	−2.398	0.017048	*
RSCPU_HEK_ERX	0.75627	0.28138	2.688	0.00756	**
Lag2	0.19206	0.05397	3.559	0.000428	***
Lag1	0.38437	0.05391	7.13	6.46E-12	***

**Figure 6:**
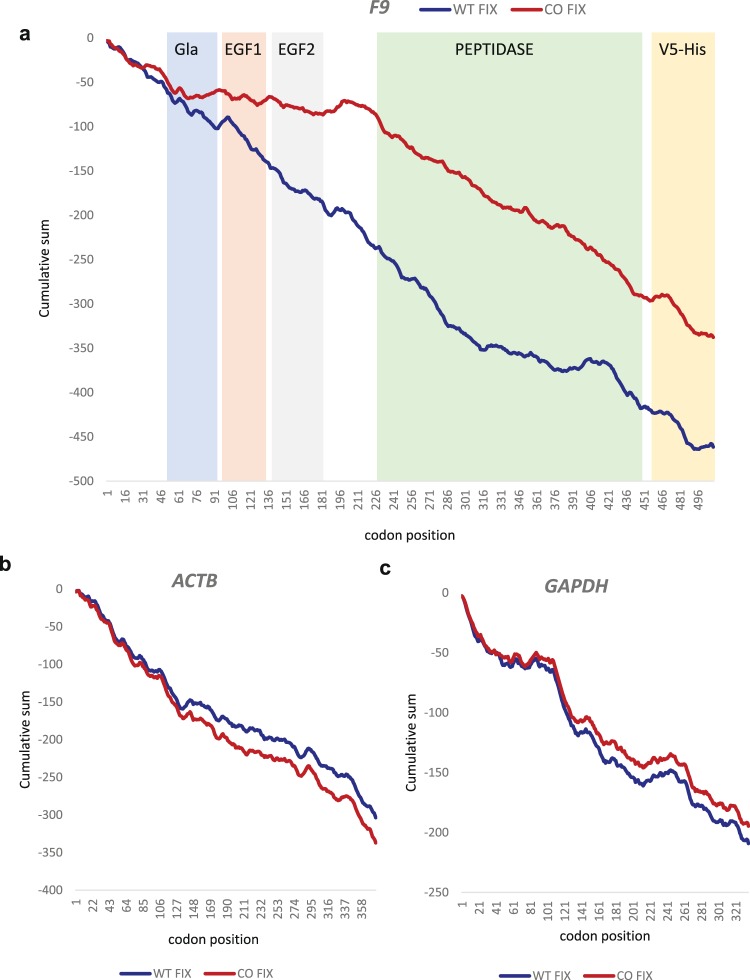
Statistical analysis of translation kinetics highlights differences in the two *F9* variants at the Gla-EGF1-EGF2 domains. Cumulative sum of (**a**) ln(RPF)*F9* values. (**b**) ln(RPF)*ACTB* values and (**c**) ln(RPF)*GAPDH* from the WT FIX and CO FIX expressing clones. The average of 3 experiments are shown.

Next, we examined whether there are any significant and systematic differences between WT and CO *F9* ribosome profiles. Although there were no significant differences in the overall translation efficiency of the two *F9* variants (Fig. 5b,c), we sought to determine whether there are differences in certain regions. Thus, we examined the cumulative sum of the normalized ln (RPF) *F9* (Fig. 6a). This type of analysis highlighted that before the start of EGF1 domain the curve for the WT translation continues to decrease linearly (similar to a Gaussian random variable, i.e., “white noise”), while the curve for CO variant does not show the same trend and doesn’t decrease as steeply until after the end of EGF2 domain. The Kolmogorov-Smirnov test yields a p-value = 3.65e-05, indicating that the two curves are significantly different. We performed the same analysis on two control genes (*ACTB* and *GAPDH*), without finding any significant difference (p-value_ACTB_ = 0.21 and p-value_GAPDH_ = 0.016; Fig. 6b,c), emphasizing that the differences observed in the WT and CO *F9* translation are not random.

## Discussion

Once considered inconsequential (and called “silent”), synonymous mutations are now accepted as substantial contributors to the cellular processes determining protein structure and function^37,52^. In the last decade there has been an exponential increase in published research on the mechanisms by which synonymous mutations influence protein folding^17,18,22^, the clinical consequences of synonymous mutations^19,20,23,27^, and the role of synonymous mutations in evolutionary biology^3,4^. Most of the studies linking synonymous mutations to disease have focused on single (or at most a few) nucleotide substitutions. Generating a codon-optimized gene, on the other hand, involves a large-scale alteration of the sequence. In this study we have used as a model protein human FIX, a marketed therapeutic protein that is also the focus of gene therapy clinical trials.

The knowledge gap that we aim to fill is the impact of synonymous mutations in the effectiveness and safety of codon-optimized proteins. Proteins generated using codon-optimized constructs are used almost universally in research and in the manufacture of therapeutic proteins^53^. However, little is understood about how proteins synthesized using codon-optimization compare to those obtained from the WT gene. This is an important area of investigation because although codon optimized proteins offer advantages to the biopharmaceutical industry they also carry potential risks as their modified nucleotide sequences may have unpredictable effects on the structure and function of proteins^14^. It is thus necessary to have well qualified methods that are fit-for-purpose and can be used to evaluate the risk of codon optimization during drug development and manufacture.

By codon optimizing human *F9*, through one of the early GeneArt algorithms, we made multiple changes in the sequence beyond the replacement of rare codons with common ones. These included increases in GC content and frequent codon pairs and a decrease in mRNA MFE and changes in equilibrium base-pairing probability (Fig. 1 and Supplemental Fig. S1 and S2). Any of these changes, which are interconnected, may be involved in the increased expression that we observed in the CO FIX. There is evidence to suggest that codon optimization improves protein production primarily by increasing mRNA transcription^32^. An increase in GC content was proposed as a possible explanation for this effect^54,55^. On the other hand, there are reports, in yeast, arguing that an increase in codon optimality leads to increased mRNA stability resulting in elevated protein expression^56^. Similarly, Chen *et al*.^57^ reported that, in yeast, CAI and mRNA levels are positively correlated and that this is, at least partially, due to the effects of codon usage on mRNA stability. A correlation between mRNA levels and GC content at position 3 of the codon (GC3) was also reported, highlighting that codon usage and GC content are often associated^57^. In agreement with these published data, here, we show that codon optimization of *F9* led to increased mRNA levels, in cells with the same plasmid DNA copy numbers (Fig. 2). On the contrary, we did not observe a significant increase in translation rate as indicated by the *in vitro* translation data (Fig. 5c), the ribosome profiling (translation efficiency, Fig. 5b) and by comparing the mRNA levels in cells expressing similar protein levels (Fig. 3). It should however be noted that unaltered translational efficiency despite substantially stronger mRNA secondary structure may suggest a positive effect of codon optimization on translational efficiency.

In comparing the WT and CO FIX molecules our goal was to determine whether altering the sequence, and resultant translational kinetics, affects conformation. Although increased expression is the desired outcome of codon optimization, in this study it was necessary to control over-expression to avoid the confounding influence of stress on the quality control machinery of the cell^45^. Thus, we were diligent in selecting WT and CO FIX-expressing cell populations which had very similar mRNA and proteins levels (Fig. 3).

We probed the conformation of the FIX variants using an array of bioanalytical techniques. The CO FIX that was investigated had similar specific activity levels to the WT protein (Fig. 4a); however, a lower concentration of hemophilia B serum, with anti-FIX antibodies, was required to inhibit its activity by 50%, compared to the WT FIX (Fig. 4b). This result may have led to an altered conformation and could have implications regarding the immune response against biotherapeutics.

Robust techniques to routinely and inexpensively determine differences in the tertiary and quaternary structures of protein therapeutics are a key unmet need. Aptamers have emerged as an important tool to probe structural changes between proteins that are nominally the same, e.g. a biosimilar and an innovator, during the manufacture of different batches of a drug etc. In a recent study a panel of aptamers was used to detect subtle differences in various thrombin products^50^, six aptamers specific to the therapeutic antibody, rituximan were used to detected conformational differences between the originator/biosimilar and a unlicensed copy product^58^ and aptamers have been used to detect and monitor bioactive peptides and proteins in foods^59^. Here we used next generation aptamers (SOMAmers) and were able to detect significant conformational differences between WT and CO FIX (Fig. 4c). Finally, we exploited limited proteolysis mediated by cathepsin and demonstrated differential digestion patterns (Fig. 4d). The variations in the digestion of WT and CO FIX likely arise from differences in conformation which either limit or favor accessibility of the proteolytic enzyme to the specific cleavage sites. Taken together these three different lines of evidence indicate that there are conformational differences between WT and CO FIX.

The drastically different translation kinetics of the two variants, as observed through ribosome profiling, provide a reasonable cause for the observed conformation differences. Studies suggesting that translational pauses have a role in modulating protein conformation have been reported since the 90’s in *Escherichia coli*^60,61^. Since then, an association between translational kinetics and protein folding has been shown in yeast and in human cells^52^. There have been several attempts to mechanistically study how ribosomal pausing may affect protein folding^62^ and unravel what dictates the time that the ribosome spends on a particular codon^63^ but there has been limited consensus. In yeast, the relative decoding time of each codon has been estimated and frequent codons have been generally shown to be decoded faster than rare codons. Furthermore, AT-rich codons were shown to be translated faster than GC-rich codons^63^. It is, however, uncertain if this applies in higher eukaryotes and particularly humans.

In our statistical analysis we were not able to detect a significant association between codon rarity and ribosomal pausing, within the *F9* gene (Table 1 and Supplementary Table 1), however this does not dictate that codon rarity and translational speed are not associated on a global level. Instead it may suggest that the association is complex and probably includes several confounding parameters. Given that FIX was expressed in HEK293T cells we also considered codon frequency in the context of HEK293T transcriptomic data. Similarly, we did not find an association between tRNA abundance and decoding times in the entire *F9* gene (Table 1 and Supplementary Table 1). Codon pair usage, which, in some systems, has been shown to be critical in enhancing protein expression^64^ was investigated, as well. Of note, increased protein expression, mediated by codon pair optimization, was reported to occur in the absence of increased mRNA levels^64^, and there is evidence suggesting that codon pair usage changes may be involved in human diseases^21^. Nevertheless, codon pair frequencies did not correlate with the ribosome profiling data (Table 1 and Supplemental Table 1). A correlation between MFE values and ribosome profiling data was detected but appeared to be gene specific; it only reached statistical significance for the WT *F9* gene but not for the control genes, *GAPDH* and *ACTB* (Table 1 and Supplementary Table 1). Importantly, mRNA MFE has been shown to be involved in disease phenotype for a limited number of genes including *F9*;^65^ however, there were not enough data to generalize this finding. The only parameter that appeared to consistently correlate with the decoding time of a codon was the decoding time of the previous two codons, suggesting that clusters of slow codons may be important in translational kinetics (Table 1 and Supplementary Table 1).

To further explore whether clusters of slowly translated codons may have a role in driving the conformational differences of the CO FIX, we examined the cumulative sum of the normalized ribosome profiling data. In this analysis we identified systematic differences between the translation kinetics of the two *F9* gene variants (Fig. 6). Interestingly, we determined that the two variants diverge from each other in their translation patterns mid-way through the Gla domain, the divergence continues until after the EGF2 domain has been translated. The Gla domain, where several glutamic acids are γ-carboxylated is critical for the function of FIX. The EGF domains, on the other hand, are crucial for the three-dimensional structure of the protein as they contain most of the cysteines involved in bridge formation. It is plausible that differences in the translational kinetics of these domains may be responsible for the conformational changes that we observed between WT and CO FIX.

Codon optimization can have unpredictable results; codon optimized proteins are not always identical to their wild-type counterparts^53^, the benefits of increased expression, however, have made this technique a common practice both in academic and biotechnology settings. In this report we characterized our model gene/protein starting from the nucleotide sequence features, to the kinetics of its translation and to the conformation of its domains. Importantly, we have appropriated a range of bioanalytical techniques that may be used to identify potential differences between the WT and CO variants, enabling the evaluation of codon-optimized protein therapeutics. In addition, this holistic approach may facilitate prediction of the effects of codon optimization and may enable the development of safer and/or more efficacious biotherapeutics.

## Methods

### *In silico* analyses of wild-type and codon optimized *F9* sequences

The WT (RefSeq NM_000133.3) and CO *F9* mRNA sequences were analyzed using a variety of tools. The mRNA secondary structure and stability were analyzed using NUPACK^66^ (http://www.nupack.org/, not allowing pseudoknots) software. RSCU^67^ and RSCPU^44^ were calculated as previously described. CAI was calculated as originally described by Sharp and Li^67^.

### Plasmid/vector construction

WT and CO (GeneArt) *F9* ORFs were sub-cloned into pcDNA3.1/V5-His-TOPO (Invitrogen/Life Technologies) according to manufacturer’s instructions to generate pcDNA3.1-*F9*-V5-His plasmids used in the stable transfections. Each fusion construct (WT*F9*-V5-His and CO*F9*-V5-His) was sub-cloned into a lentiviral vector pTK642 (gift from Dr. Kafri, University of North Carolina at Chapel Hill) at the Pacl/Sfil site.

### Cell cultures

Human embryonic kidney cells (HEK293T; ATCC) were grown in Dulbecco’s Modified Eagle Medium (Quality Biological, Inc) with 1% L-glutamine (Quality Biological), 1% penicillin- streptomycin (Hyclone) and 10% fetal bovine serum (Quality Biological) at 37 °C in 5% CO_2_. HEK293T were transfected with WT-*F9*- V5-His pcDNA3.1 or CO-*F9*-V5-His pcDNA3.1 and cultured in medium containing 500 ug/ml G418 to generate stably expressing cell populations. Alternatively, HEK293T cells stably expressing WT or CO FIX were established following transduction with lentiviral vectors, as previously described^68^.

An equivalent number of cells were plated in T-flasks and supplemented with 10 ng/ml of Vitamin K3, one day prior to all experiments. The culture medium was replaced with Opti-MEM Reduced Serum Medium (Life Technologies) at approximately 80–90% cell confluency and cells were harvested after an additional 24 hours of incubation. Protein concentration in cell lysates and medium was measured using the Quick Start™ Bradford (Bio-Rad) assay according to manufacturer instructions.

### DNA and RNA isolation and quantitative real time PCR (qPCR)

Genomic DNA from FIX stable expression cell lines was extracted using DNeasy Blood & Tissue Kit (Qiagen, Germantown, MD), according to manufacturer’s instructions. RNA was isolated using RNeasy Plus Mini Kit (Qiagen) following manufacturer’s instructions. Reverse transcription was carried out with High-Capacity cDNA Reverse Transcription Kit (Applied Biosystems). qPCR was performed on LightCycler 480 (Roche) using TaqMan Universal PCR Master Mix. Custom made Taqman primers and probe targeting CMV promoter sequence present in all *F9* constructs was used in the *F9* DNA copy number assay. Standard curves were generated using pTK642 plasmid ranging from 100,000 to 32 copies. The transgenic *F9* copy numbers in stable expression cell lines were calculated against standard curve using genomic DNA input ranging from 200 ng to 8 ng in qPCR reactions. Average genomic DNA yield of 6.6 pg/cell was employed in the calculation of copies per cell. TaqMan Gene Expression Assays targeted against GAPDH Hs02758991_g1) and V5-His tag sequences of FIX constructs (custom made) were used for *F9* mRNA quantitation assay. Crossing point (Cp) values were obtained and the ΔΔCp was calculated using *GAPDH* as the reference gene.

Taqman primers and probe targeting CMV promoter sequence present in all *F9* constructs.

### Confocal microscopy

The HEK293T cells stably transfected with either empty vector (pcDNA3.1/V5-His-TOPO), WT *F9* (pcDNA3.1-WT*F9*-V5-His) or CO *F9* (pcDNA3.1-CO*F9*-V5-His) were grown on poly-L-lysine coated culture dishes for 24 hours. The cells were then fixed with 4% paraformaldehyde for 10 min, followed with 0.05% Triton X for 30 min. Cells were then incubated with 5% normal donkey serum for 30 minutes at room temperature, followed with primary antibodies, mouse anti-V5, 1:100 dilution (Invitrogen) for overnight at 4 °C. Cells were then washed with PBS and incubated with the secondary antibody, Alexa488 conjugated donkey anti-mouse IgG (Molecular Probes) at a 1:250 dilution. Cells were observed by Leica TCS_SP-8 DMI6000 confocal microscope system. About 60 cells expressing FIX were analyzed for each condition using ImageJ software.

### SDS PAGE analysis

Cell culture supernatant and cell lysates were prepared for SDS PAGE analysis as described earlier^62^. Mouse monoclonal antibodies, anti-V5 (R960–25, Thermo Fisher) and anti-GAPDH (MA5–15738, Thermo Fisher) and polyclonal goat anti-mouse IgG HRP conjugate antibody (31430, Thermo Fisher) were used at 1:10,000 dilution. Densitometry analysis was performed with ImageJ software.

### Protein purification

Proteins were purified via affinity chromatography, using V5-tagged purification kit (MBL International) per manufacturer’s protocol. Briefly, Opti-MEM reduced serum media (Life Technologies) collected from FIX stable expression cells were concentrated and incubated with anti-V5 tag beads. The beads were then washed with PBS and bound FIX was eluted using V5 peptide. The purity of the protein preparation was assessed using silver staining, as previously described^23^.

### FIX activity assay and inactivation by inhibitory plasma

FIX deficient plasma supplemented with inhibitory antibodies against FIX (Affinity Biologicals) or normal human plasma obtained from NIH Blood Bank were heat inactivated at 56 °C for 30 minutes and centrifuged to remove precipitates. Purified FIX samples were incubated with serial dilutions of the plasma, at 30 °C for 1 hr. FIX activity of WT and CO FIX (20 ng/ml) after treatment with plasma was measured in three experiments by chromogenic assay using Biophen factor IX kit (Aniara). Using 4-parameter dosage response curves stratified by treatment type, half-max values were calculated for both WT and CO.

### Measurement of SOMAmer-FIX kinetic parameters with a label-free technology

The SOMAmer (Slow Off-rate Modified Aptamers) targeting human FIX, S-12X, was generated and validated by SomaLogic, Boulder CO. Affinity measurements using BioLayer Interferometry (BLI) were performed with ForteBio Octet RED96 equipped with streptavidin (SA) biosensor tips (ForteBio, Inc., Menlo Park, CA, USA). The assays were maintained at a temperature of and the speed of 30 °C and 1000 rpm respectively. Streptavidin-coated biosensor tips were pre-wet for 15 min. Then the tips were loaded with 250–500 nM of biotinylated SOMAmer (or anti-V5 antibody for control). The association (Kon) and dissociation (Kdis) were then established by transfering the biosensors for 10 mins in various concentrations of FIX dispensed in 96- microwell plates (Fisher Scientific) at a volume of 200 µl per well. Data were processed and analyzed using the Octet data analysis software version 7.0 (ForteBio). The binding profile of each SOMAmer was shown as “nm” shift. This shift is a comparison of the shift in the interference patterns of light reflected from a reference layer within the biosensor versus molecules as the bind to the biosensor tip. The results were summarized as KD which was calculated from “KD = kon/kdis”, where ka is the ‘on rate’ or association and kdis is the ‘off rate’ or dissociation.

### Cathepsin L digestion

Equal amounts of purified proteins, based on silver staining were subjected to limited proteolysis with increasing cathepsin L concentrations (Sigma Aldrich) (0.2, 0.1, 0.05, 0 ng/ml) for 3 minutes at 37 °C. Digestion was terminated by adding SDS-sample buffer and boiling the samples for 15 minutes at 100 °C. Samples were analyzed by SDS-PAGE and silver staining. Densitometry analysis was performed with imageJ software.

### *In vitro* translation

*In vitro* translation of *F9* mRNAs was performed in the presence of [^35^S]-Met following standard procedures with Rabbit Reticulocyte Lysate (RRL) system (Promega) supplemented with calf liver tRNAs as described previously^69^.

### Ribosome profiling

HEK293 cells expressing WT and CO *F9* were harvested at ~80% confluence following overnight incubation in Opti-MEM to maintain consistency with protein level processing and analyses presented in this paper. Ribosome profiling was conducted as described previously^70^ using the Illumina TruSeq Ribo Profile (Mammalian) Kit according to manufacturer’s instructions with modifications in harvest, RNA isolation/purification (isopropanol isolation used to improve the yield) and RPF size selection (~20–32 nt). During harvest, media was carefully removed, and cells were immediately flash-frozen. All equipment used from hence forth was pre-chilled. Cells were quickly scrapped into 1 ml of ice-cold lysis buffer (5X Mammalian Polysome Buffer, 10% Triton-X100, 100 mM DTT, DNase I, Nuclease-free water) and homogenized on ice by passing through a 26 G needle 10 times. Lysate was then spun at 4 °C for 10 minutes at 20,000 × g. Supernatant was aliquoted into cryovials and immediately frozen in liquid nitrogen for future use. Samples were sequenced using Illumina HiSeq2500.

Sequencing data were pre-processed by adapter trimming (FASTX Toolkit) and the removal of contaminating rRNA and tRNA sequences (bowtie version 1.0.0; parameter ‘-l 20’ was used, all other parameters default). Fragments smaller than 25 nt in length were removed from the total mRNA samples. Using TopHat version 2.0.9 (parameters ‘–no-novel-juncs -g 20’ were used, all other parameters default), RPF and total mRNA populations were aligned to a custom human transcriptome built using the GENCODE hg19 CDS and UTR annotations in addition to the WT and CO *F9* CDS flanked by 100 base pairs of vector sequence which replaced the chromosomal *F9* transcript sequence.

RPF sequences were analyzed based on fragment length (Supplemental Fig. S7b,c), alignment distribution between coding sequences (CDS) and 5′- and 3′-UTRs (Supplemental Fig. S7d), triplet periodicity and reading frame (Supplemental Fig. S7e). RPF fragments 20–22nt and 27–29 nt in length were used for further analysis with an A-site offset of 15 nucleotides. Pearson’s correlation was used to evaluate the reproducibility between samples using a common subset of moderately to highly expressed genes (reads per kilobase of transcript per million mapped reads, RPKM_CDS_ ≥ 10) (Supplemental Fig. S8).

Translational kinetics of *F9* and two housekeeping genes, *GAPDH* and *ACTB*, were further analyzed. Translation efficiency for each gene was calculated as TE = RPF(RPKM)/Total mRNA (RPKM)^29,71^. Normalized codon coverage for *F9*, *GAPDH* and *ACTB* CDSs was calculated as [#RPFs with codon X in A site/average #RPFs from CDS]. Data was then averaged across three replicates to generate final RPF coverage plots. Normalized RPF coverage for the CDSs of each gene was plotted using A-site fragment density per codon. Of note, the CDS for WT and CO *F9* included a portion of the common vector sequence (codons 463–509).

### Statistical analysis of ribosome profiling

The RPF data were transformed using the Box-Cox variance-stabilizing transformation to obtain normal distributions. For *F9, GAPDH*, and *ACTB*, we created a linear regression model using the normalized RPF as a response (Supplemental Table S1), using the following explanatory variables: RSCU-Genome (RSCU values calculated from *Homo sapiens* genomic codon usage statistics^1^), RSCU-HEK-SRR (RSCU values calculated from HEK transcriptome codon usage statistics (https://www.ncbi.nlm.nih.gov/sra/?term=SRR5922096), RSCU-HEK_ERX (RSCU values calculated from HEK transcriptome codon usage statistics (https://www.ncbi.nlm.nih.gov/sra/?term=ERX2016843), RSCPU-Genome (RSCPU values calculated from *Homo sapiens* genomic codon usage statistics^44^), RSCPU-HEK-SRR (RSPCU values calculated from HEK transcriptome codon usage statistics (https://www.ncbi.nlm.nih.gov/sra/?term=SRR5922096), RSCPU-HEK_ERX (RSCPU values calculated from HEK transcriptome codon usage statistics (https://www.ncbi.nlm.nih.gov/sra/?term=ERX2016843), tRNA (tRNA levels derived from published data^72^), MFE (MFE values calculated for 150 nucleotides centered around the codon located at the A site), pair-prob (base-pairing probability was computed using the Vienna RNA website and the three constituent nucleotides were averaged for each codon), Lag1 (RPF values at the A + 1 site), Lag2 (RPF values at the A + 2 site), and Lag3 (RPF values at the A + 3 site). Then, we ran step-wise model selection on the linear regression model. We used the “stepAIC” function in R, with its default settings. The function chooses a model based on the Aikake Information Criterion, AIC (Table 1).

To test for systematic differences between WT and CO *F9* ribosome profiles, we first performed a Box-Cox transformation to normalize the data, because the raw data is very skewed. Then, we used a model which accounts for the strong correlations between adjacent codons. Performing a two-group comparison separately for each codon is inadequate, since it completely ignores the spatial correlation which is very clearly present. The cumulative sum of the transformed RPF was used to account for the spatial correlation, and the resulting data was compared with data from a simulated Gaussian process, so as to test the null hypothesis that the observed data (in terms of the cumulative sum of the Box-Cox transformed data) did not differ from a random sequence (“white noise”). We used the cumulative sum data to also test whether the two *F9* variants differed. To test the hypothesis formally, we used the Kolmogorov-Smirnov test which is commonly used to test for difference between probability distribution functions. The test for comparing *F9* WT and CO yielded a p-value = 3.65e-05, while comparing the two groups in the control genes gave non-significant results.

## Supplementary information

Supplementary Figures and Table

## Data Availability

The datasets generated during and analyzed during the current study are available from the corresponding author on reasonable request.
