# Supplementary material for: Effects of codon optimization on coagulation factor IX translation and structure: Implications for protein and gene therapies
Source: Sci Rep. 2019 Oct 29;9:15449. doi: 10.1038/s41598-019-51984-2 (PMC6820528; doi:10.1038/s41598-019-51984-2)
Supplement: Supplementary file 1 — Supplementary Figures and Table [file 41598_2019_51984_MOESM1_ESM.pdf]

Effects of codon optimization on coagulation factor IX translation and structure: Implications for protein and gene therapies

Aikaterini Alexaki [a]*, Gaya K. Hettiarachchi [a]*, John C. Athey [a], Upendra K. Katneni [a], Vijaya Simhadri[a], Nobuko Hamasaki-Katagiri [a], Puja Nanavaty[b], Brian Lin [a], Kazuyo Takeda[a], Darón Freedberg[a], Dougald Monroe[c], Joseph R. McGill [a], Robert Peters[d], Jacob M. Kames[a], David D. Holcomb[a], Ryan C. Hunt [a], Zuben E. Sauna[a], Amy Gelinas[e], Nebojsa Janjic[e], Michael DiCuccio[f], Haim Bar[g], Anton A. Komar[b], and Chava Kimchi-Sarfaty[a]

[a] Center for Biologics Evaluation and Research, Food and Drug Administration, Silver Spring, MD, USA

[b] Center for Gene Regulation in Health and Disease, Cleveland State University, Cleveland, OH, USA

[c] University of North Carolina at Chapel hill, Chapel hill, NC, USA

[d] Bioverativ, Cambridge, MA, USA

[e] SomaLogic, Inc., Boulder, CO, USA

[f] National Center of Biotechnology Information, National Institutes of Health, Bethesda, MD, USA

[g] Department of Statistics, University of Connecticut, Storrs, CT, USA

Corresponding author:

Chava.kimchi-sarfaty@fda.hhs.gov; Division of Plasma Protein Therapeutics, Office of Tissue and Advanced Therapies, Center for Biologics Evaluation and Research, Food and Drug Administration, Silver Spring, USA

*Authors AA and GKH contributed equally

Supplemental Figure S1. Comparison of the nucleotide sequences of codon-optimized and wild type *F9* variants. Nucleotide changes between the two sequences are indicated in red, while codon changes are highlighted in yellow.

```
CO    1 ATGCAGAGAGTCAACATGATTATGGCTGAGTCACCTGGGCTGATTACTAT
WT    1 ATGCAGCGCGTGAACATGATCATGGCAGAATCACCAGGCCTCATCACCAT
          M  Q  R  V  N  M  I  M  A  E  S  P  G  L  I  T  I

CO   51 TTGCCTGCTGGGCTACCTGCTGTCCGCCGAGTGTACCGTGTTCCTGGA
WT   51 CTGCCTTTTAGGATATCTACTCAGTGCTGAATGTACAGTTTTTCTTGA
           C  L  L  G  Y  L  L  S  A  E  C  T  V  F  L  D

CO   99 CCATGAGAACGCAAATAAGATCCTGAACAGGCCCAAAAGATATAATAGTG
WT   99 TCATGAAAACGCCAACAAAATTCTGAATCGGCCAAAGAGGTATAATTCAG
          H  E  N  A  N  K  I  L  N  R  P  K  R  Y  N  S  G

CO  149 GGAAGCTGGAGGAATTTGTGCAGGGCAACCTGGAGAGAGAATGCATGGAG
WT  149 GTAAATTGGAAGAGTTTGTTCAAGGGAACCTTGAGAGAGAATGTATGGAA
           K  L  E  E  F  V  Q  G  N  L  E  R  E  C  M  E

CO  199 GAAAAGTGTAGCTTCGAGGAAGCCCGCGAGGTGTTTGAAAATACAGAGCG
WT  199 GAAAAGTGTAGTTTTGAAGAAGCACGAGAAGTTTTTGAAAACACTGAAAG
        E  K  C  S  F  E  E  A  R  E  V  F  E  N  T  E  R

CO  249 AACCACAGAGTTCTGGAAGCAGTATGTGGACGGCGATCAGTGCGAGAGCA
WT  249 AACAACTGAATTTTGGAAGCAGTATGTTGATGGAGATCAGTGTGAGTCCA
          T  T  E  F  W  K  Q  Y  V  D  G  D  Q  C  E  S  N

CO  299 ACCCCTGTCTGAATGGCGGAAGTTGCAAAGACGATATCAACTCATACGAA
WT  299 ATCCATGTTTAAATGGCGGCAGTTGCAAGGATGACATTAATTCCTATGAA
            P  C  L  N  G  G  S  C  K  D  D  I  N  S  Y  E

CO  349 TGCTGGTGTCCTTTCGGGTTTGAAGGCAAAAATTGCGAGCTGGACGTGAC
WT  349 TGTTGGTGTCCCTTTGGATTTGAAGGAAAGAACTGTGAATTAGATGTAAC
        C  W  C  P  F  G  F  E  G  K  N  C  E  L  D  V  T

CO  399 ATGTAACATTAAGAATGGACGGTGCGAGCAGTTTTGTAAAAACTCTGCCG
WT  399 ATGTAACATTAAGAATGGCAGATGCGAGCAGTTTTGTAAAAATAGTGCTG
          C  N  I  K  N  G  R  C  E  Q  F  C  K  N  S  A  D

CO  449 ATAATAAGGTGGTGTGCAGCTGTACTGAAGGATATCGCCTGGCTGAGAAC
WT  449 ATAACAAGGTGGTTTGCTCCTGTACTGAGGGATATCGACTTGCAGAAAAC
           N  K  V  V  C  S  C  T  E  G  Y  R  L  A  E  N

CO  499 CAGAAGTCCTGCGAACCAGCAGTGCCCTTCCCTTGTGGGAGGGTGAGCGT
WT  499 CAGAAGTCCTGTGAACCAGCAGTGCCATTTCCATGTGGAAGAGTTTCTGT
        Q  K  S  C  E  P  A  V  P  F  P  C  G  R  V  S  V

CO  549 CTCCCAGACTTCAAAACTGACCAGAGCAGAGACAGTGTTTCCCGACGTGG
WT  549 TTCACAAACTTCTAAGCTCACCCGTGCTGAGACTGTTTTTCCTGATGTGG
          S  Q  T  S  K  L  T  R  A  E  T  V  F  P  D  V  D

CO  599 ATTACGTCAACAGCACTGAGGCCGAAACCATCCTGGACAACATTACTCAG
WT  599 ACTATGTAAATTCTACTGAAGCTGAAACCATTTTGGATAACATCACTCAA
           Y  V  N  S  T  E  A  E  T  I  L  D  N  I  T  Q

CO  649 TCTACCCAGAGTTTCAATGACTTTACTCGGGTGGTCGGGGGCGAGGATGC
WT  649 AGCACCCAATCATTTAATGACTTCACTCGGGTTGTTGGTGGAGAAGATGC
        S  T  Q  S  F  N  D  F  T  R  V  V  G  G  E  D  A

CO  699 TAAACCAGGCCAGTTCCCCTGGCAGGTGGTCCTGAACGGAAAGGTGGATG
WT  699 CAAACCAGGTCAATTCCCTTGGCAGGTTGTTTTGAATGGTAAAGTTGATG
          K  P  G  Q  F  P  W  Q  V  V  L  N  G  K  V  D  A

CO  749 CATTTTGCGGAGGGTCTATCGTGAATGAGAAATGGATTGTCACCGCCGCT    798
WT  749 CATTCTGTGGAGGCTCTATCGTTAATGAAAAATGGATTGTAACTGCTGCC    798
           F  C  G  G  S  I  V  N  E  K  W  I  V  T  A  A

CO  799 CACTGCGTGGAAACCGGAGTCAAGATCACAGTGGTCGCTGGGGAGCACAA    848
WT  799 CACTGTGTTGAAACTGGTGTTAAAATTACAGTTGTCGCAGGTGAACATAA    848
        H  C  V  E  T  G  V  K  I  T  V  V  A  G  E  H  N

CO  849 CATTGAGGAAACAGAACATACTGAGCAGAAGCGGAATGTGATCCGCATCA    898
WT  849 TATTGAGGAGACAGAACATACAGAGCAAAAGCGAAATGTGATTCGAATTA    898
          I  E  E  T  E  H  T  E  Q  K  R  N  V  I  R  I  I

CO  899 TTCCTCACCATAACTACAATGCAGCCATCAACAAATATAATCATGACATT    948
WT  899 TTCCTCACCACAACTACAATGCAGCTATTAATAAGTACAACCATGACATT    948
           P  H  H  N  Y  N  A  A  I  N  K  Y  N  H  D  I

CO  949 GCCCTGCTGGAACTGGATGAGCCTCTGGTGCTGAACAGCTACGTCACTCC    998
WT  949 GCCCTTCTGGAACTGGACGAACCCTTAGTGCTAAACAGCTACGTTACACC    998
        A  L  L  E  L  D  E  P  L  V  L  N  S  Y  V  T  P

CO  999 AATCTGCATTGCTGACAAAGAGTATACCAATATCTTCCTGAAGTTTGGAT   1048
WT  999 TATTTGCATTGCTGACAAGGAATACACGAACATCTTCCTCAAATTTGGAT   1048
          I  C  I  A  D  K  E  Y  T  N  I  F  L  K  F  G  S

CO 1049 CAGGGTACGTGAGCGGCTGGGGAAGAGTCTTCCACAAGGGCAGGAGC-GC   1097
WT 1049 CTGGCTATGTAAGTGGCTGGGGAAGAGTCTTCCACAAAGGGA-GATCAGC   1097
           G  Y  V  S  G  W  G  R  V  F  H  K  G  R   S  A

CO 1098 CCTGGTGCTCCAGTATCTGCGAGTGCCTCTGGTCGATCGAGCTACCTGTC   1147
WT 1098 TTTAGTTCTTCAGTACCTTAGAGTTCCACTTGTTGACCGAGCCACATGTC   1147
          L  V  L  Q  Y  L  R  V  P  L  V  D  R  A  T  C  L

CO 1148 TGAGGTCTACCAAGTTTACAATCTACAACAACATGTTCTGCGCTGGGTTT   1197
WT 1148 TTCGATCTACAAAGTTCACCATCTATAACAACATGTTCTGTGCTGGCTTC   1197
           R  S  T  K  F  T  I  Y  N  N  M  F  C  A  G  F

CO 1198 CACGAGGGAGGACGAGACTCCTGTCAGGGCGATTCTGGGGGCCCACATGT   1247
WT 1198 CATGAAGGAGGTAGAGATTCATGTCAAGGAGATAGTGGGGGACCCCATGT   1247
        H  E  G  G  R  D  S  C  Q  G  D  S  G  G  P  H  V

CO 1248 GACAGAGGTCGAAGGCACCAGCTTCCTGACTGGCATCATTTCCTGGGGAG   1297
WT 1248 TACTGAAGTGGAAGGGACCAGTTTCTTAACTGGAATTATTAGCTGGGGTG   1297
          T  E  V  E  G  T  S  F  L  T  G  I  I  S  W  G  E

CO 1298 AGGAATGTGCAATGAAGGGAAAATACGGGATCTACACCAAAGTGAGCCGC   1347
WT 1298 AAGAGTGTGCAATGAAAGGCAAATATGGAATATATACCAAGGTATCCCGG   1347
           E  C  A  M  K  G  K  Y  G  I  Y  T  K  V  S  R

CO 1348 TATGTGAACTGGATCAAGGAAAAAACCAAACTGACC   1383
WT 1348 TATGTCAACTGGATTAAGGAAAAAACAAAGCTCACT   1383
        Y  V  N  W  I  K  E  K  T  K  L  T
```

Supplemental Figure S2. In silico analyses of mRNA folding and stability for the WT and CO F9 coding sequence. Predicted MFE values and structures are shown for 37°C based on NUPACK algorithm (www.nupack.org)

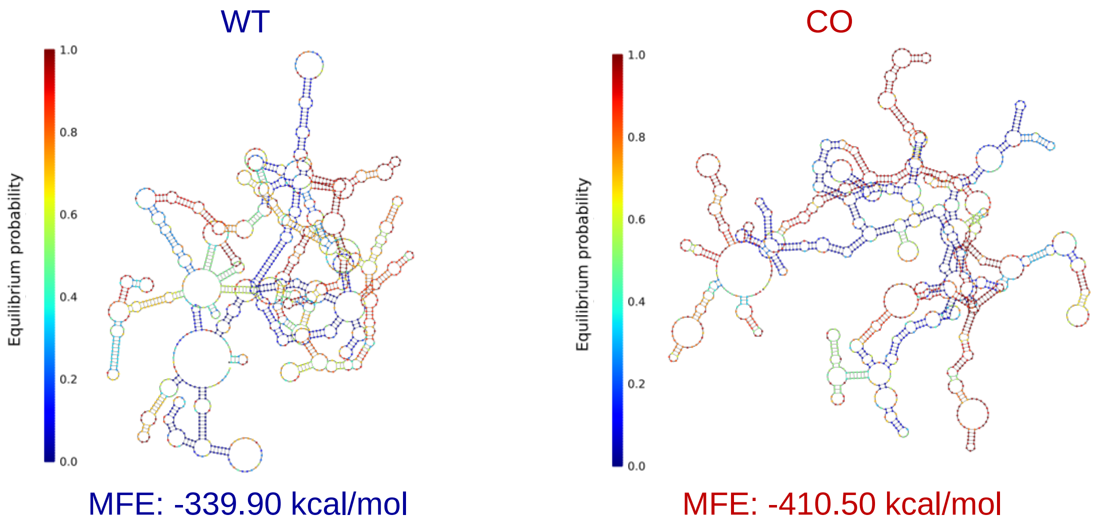

Supplemental Figure S3. FIX expression is higher in HEK293T cells stably transfected with wild-type vs. codon-optimized *F9.* Cell supernatant (concentrated medium) and lysate was collected and analyzed by immunoblotting. Membranes were stained with anti-V5 or anti-GAPDH. Multiple exposures of film are shown to demonstrate FIX (top image) and GAPDH (bottom image) at appropriate contrast. In the medium lanes, 5 µg of protein were loaded per lane. In the lysate lanes, 10 µg of protein were loaded per lane. Portions of these images were used to create Figure 2b.

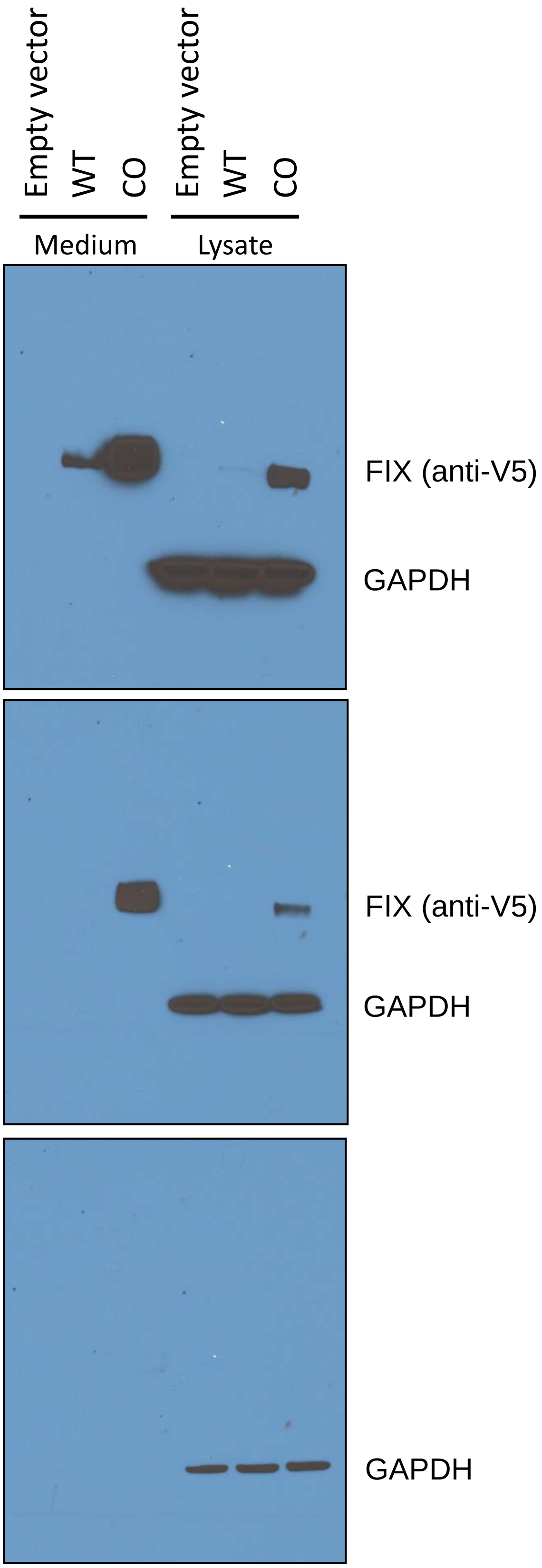

Supplemental Figure S4. RPF densities along the WT and CO *F9* mRNA for three replicates show reproducibility of results.

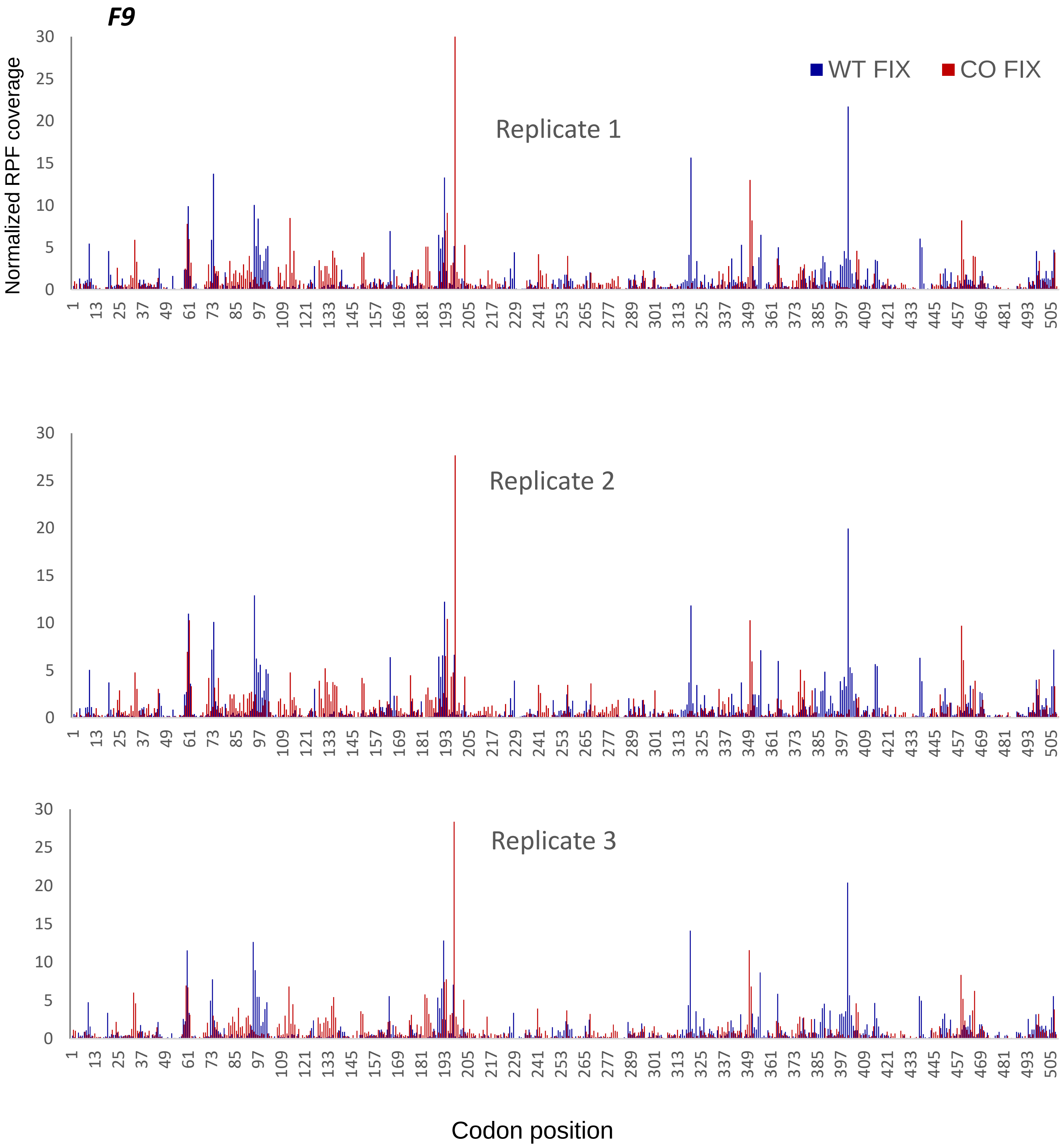

Supplemental Figure S5. *GAPDH* is translated with very similar kinetics in the wild type and codon-optimized expressing cell lines. RPF densities along the G*APDH* mRNA are shown as average of 3 experiments and in each individual ribosome profiling experiment.

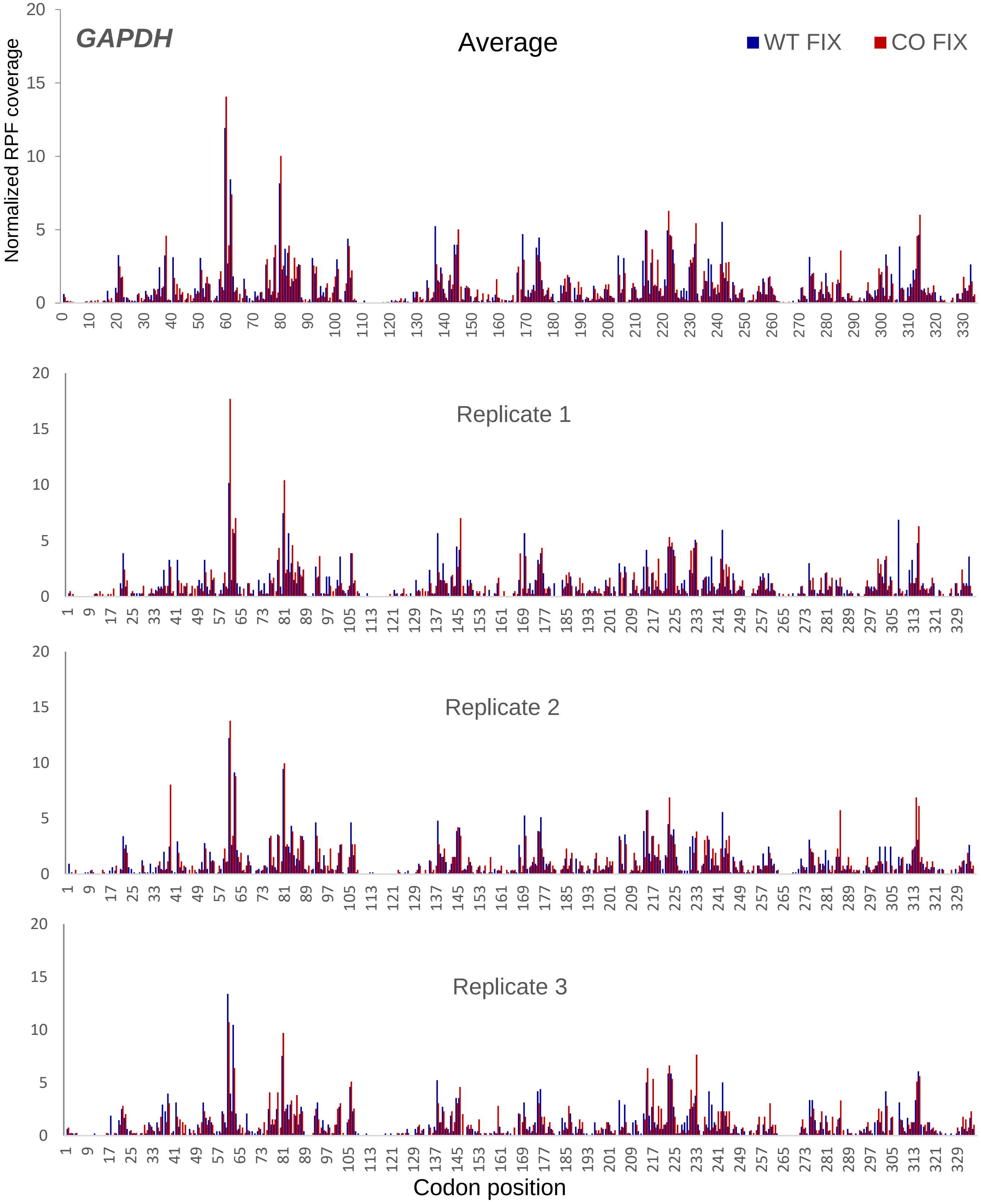

Supplemental Figure S6. *ACTB* is translated with very similar kinetics in the wild type and codon-optimized expressing cell lines. RPF densities along the *ACTB* mRNA are shown as average of 3 experiments and in each individual ribosome profiling experiment.

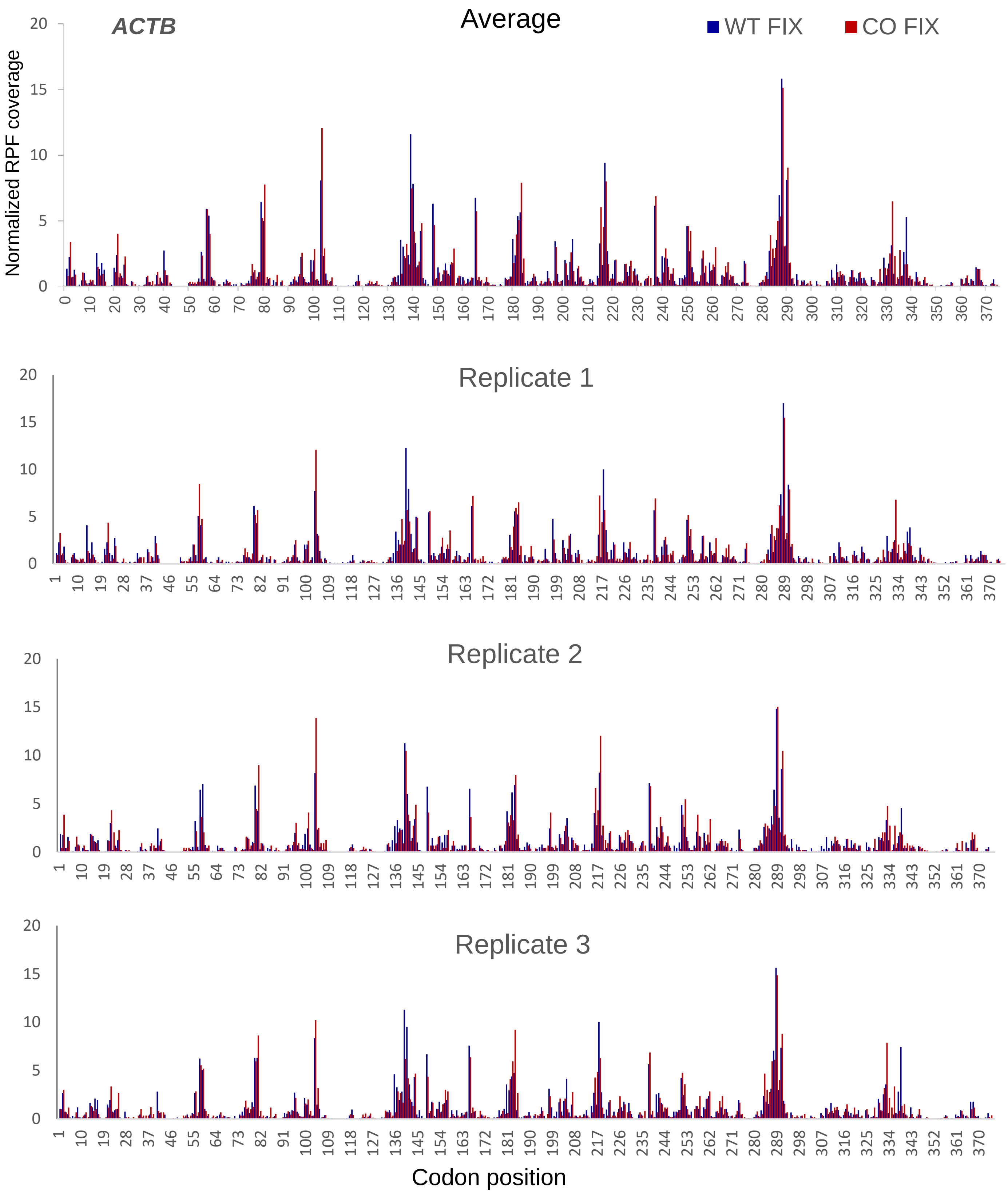

Supplemental Figure S7. Multiple sets of controls show consistency in ribosome profiling experiments and analysis (a) Transcript and RPF abundance for *F9*, *GAPDH* and *ACTB* in the WT and CO FIX expressing cell lines. The average of 3 experiments was plotted. (b) RPF size distribution of all reads in the WT and CO FIX expressing cell lines. (c) *F9* RPF size distribution in the WT and CO FIX expressing cell lines. (d) RPF sizes 20-22 and 27-29nt mapped 5'UTR, CDS and 3'UTR regions, (c) triplet periodicity of RPF fragment sizes 20-22 and 27-29 nucleotides, the average of 3 experiments was plotted, s.e.m. are shown.

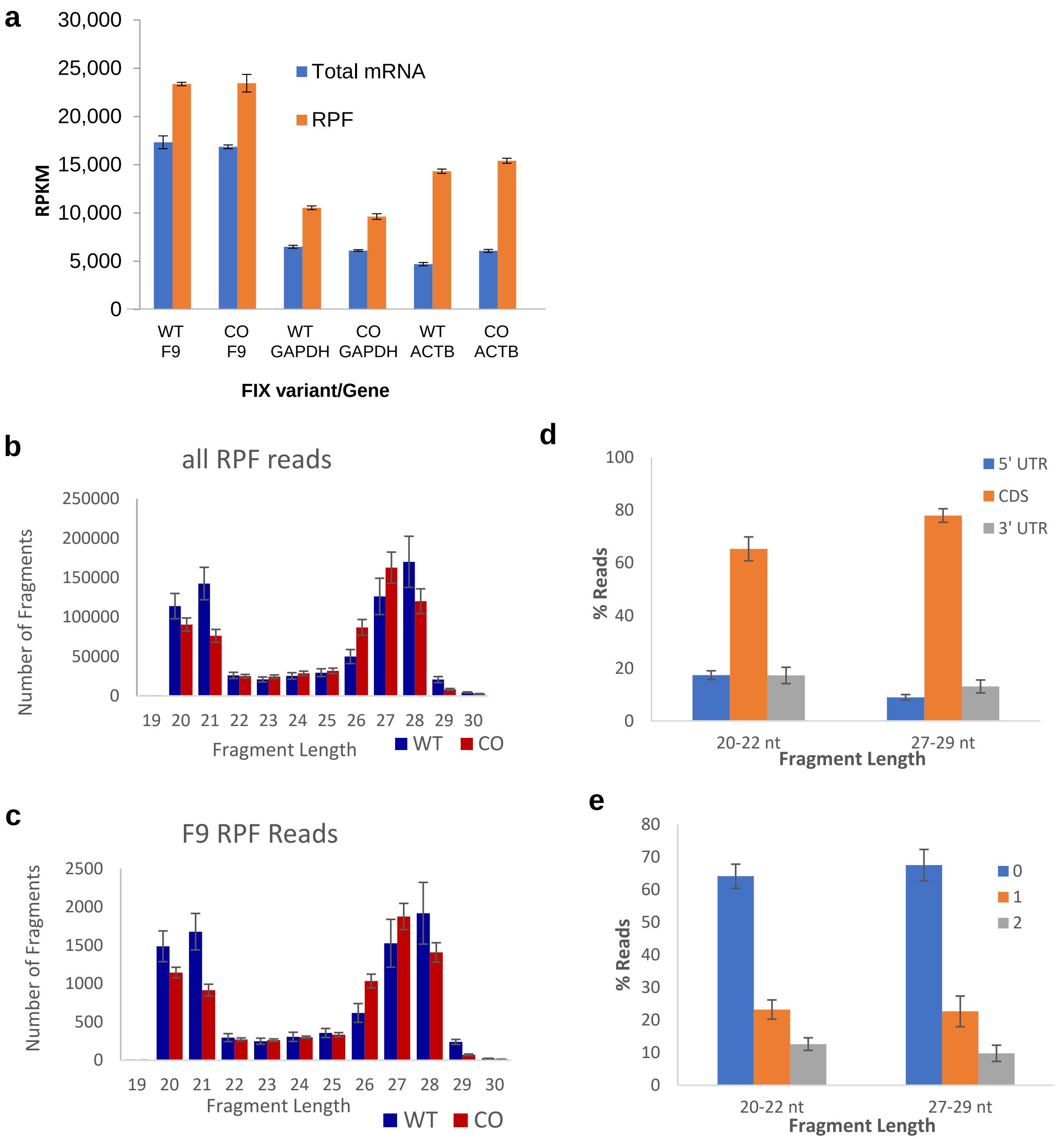

Supplemental Figure S8. Reproducibility of ribosome profiling experiments. Pearson's correlation for (a) RPFs and (b) total mRNA between two representative experiments.

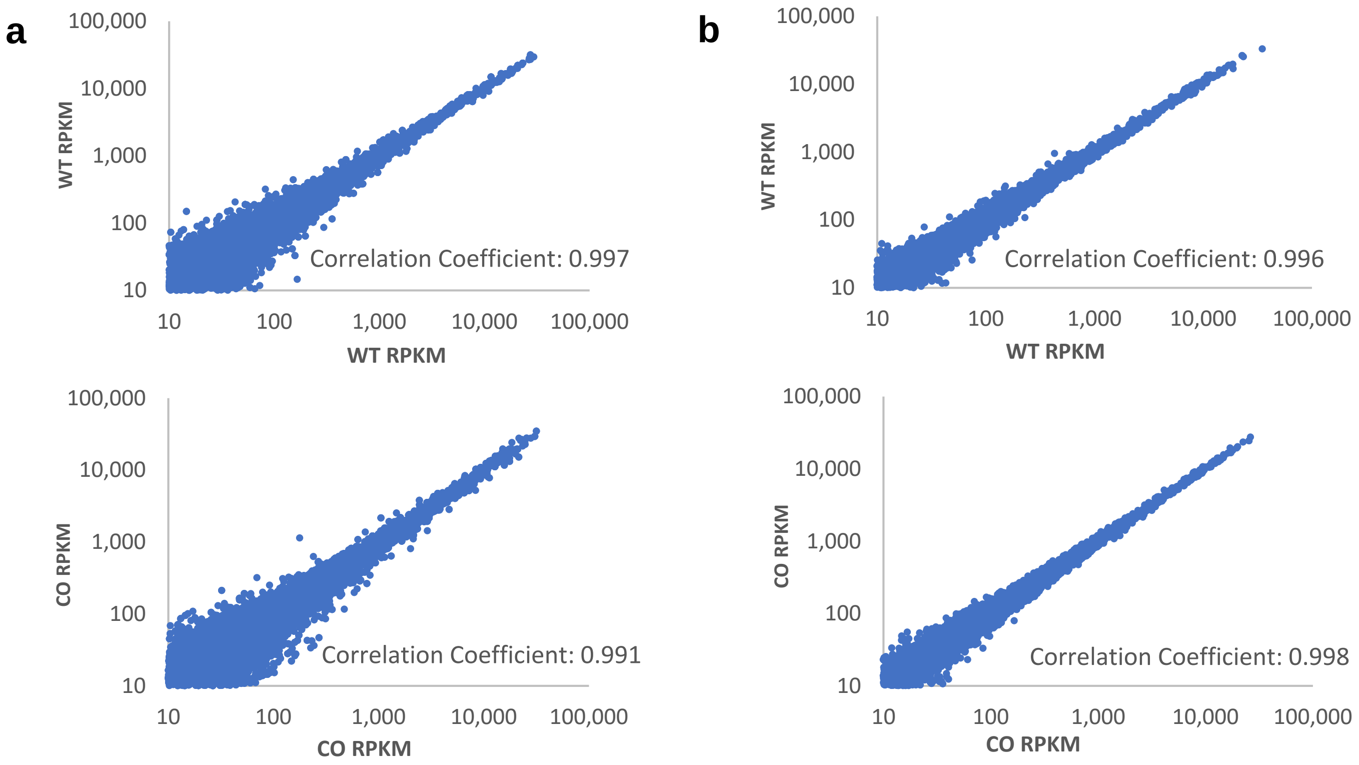

Supplemental Table 1. In a multiple linear regression model, the ribosome occupancy of adjacent codons is the only consistently significant predictor of ribosome occupancy of a codon.

	Estimate	Std. Error	t-value	Pr(>|t|)	Significance
WT *F9*					
(Intercept)	-1.05664	0.50251	-2.103	0.036	*
RSCU_Genome	0.03087	0.89716	0.034	0.9726	
RSCU_HEK_SRR	0.55596	1.06783	0.521	0.6028	
RSCU_HEK_ERX	-0.44406	1.23987	-0.358	0.7204	
RSCPU_Genome	-0.49032	0.30636	-1.6	0.1101	
RSCPU_HEK_SRR	-0.39351	0.40322	-0.976	0.3296	
RSCPU_HEK_ERX	0.73974	0.4682	1.58	0.1148	
tRNA	-4.70107	3.00585	-1.564	0.1185	
MFE_151	-0.02097	0.0105	-1.997	0.0464	*
Pair-prob	0.22075	0.16498	1.338	0.1815	
Lag3	0.05156	0.04475	1.152	0.2498	
Lag2	0.03957	0.04945	0.8	0.4239	
Lag1	0.47339	0.04486	10.554	<2e-16	***
CO *F9*					
(Intercept)	-0.428561	0.470664	-0.911	0.362978	
RSCU_Genome	0.987203	0.686076	1.439	0.150808	
RSCU_HEK_SRR	-0.510807	0.884671	-0.577	0.563933	
RSCU_HEK_ERX	-0.562106	1.060021	-0.53	0.596157	
RSCPU_Genome	-0.100063	0.230617	-0.434	0.664555	
RSCPU_HEK_SRR	0.012383	0.335691	0.037	0.970589	
RSCPU_HEK_ERX	0.119879	0.396397	0.302	0.762458	
tRNA	-3.330872	2.315622	-1.438	0.150943	
MFE_151	-0.007955	0.008367	-0.951	0.342211	
Pair-prob	-0.130687	0.200669	-0.651	0.515183	
Lag3	-0.02156	0.045746	-0.471	0.637641	
Lag2	0.177367	0.048099	3.688	0.000252	***
Lag1	0.390892	0.045033	8.68	<2e-16	***
ACTB					
(Intercept)	-1.404031	0.545822	-2.572	0.0105	*
RSCU_Genome	-0.310705	0.916587	-0.339	0.7348	
RSCU_HEK_SRR	-0.796966	1.242992	-0.641	0.5218	
RSCU_HEK_ERX	1.025136	1.514433	0.677	0.4989	
RSCPU_Genome	-0.100309	0.265983	-0.377	0.7063	
RSCPU_HEK_SRR	-0.075492	0.43977	-0.172	0.8638	
RSPCU_HEK_ERX	0.273948	0.478317	0.573	0.5672	
tRNA	1.554097	2.632387	0.59	0.5553	
MFE_151	-0.008856	0.007942	-1.115	0.2655	
Pair-prob	0.642673	0.26481	2.427	0.0157	*
Lag3	0.107027	0.052224	2.049	0.0411	*
Lag2	0.05047	0.057602	0.876	0.3815	
Lag1	0.442478	0.052295	8.461	6.64E-16	***
GAPDH					
(Intercept)	-0.149095	0.462448	-0.322	0.74736	
RSCU_Genome	1.409989	0.828001	1.703	0.08955	.
RSCU_HEK_SRR	-1.172997	1.265043	-0.927	0.3545	
RSCU_HEK_ERX	-0.093217	1.421276	-0.066	0.94775	
RSCPU_Genome	-0.652895	0.287641	-2.27	0.02388	*
RSCPU_HEK_SRR	-0.4377	0.438042	-0.999	0.31844	
RSCPU_HEK_ERX	1.113346	0.497467	2.238	0.0259	*
tRNA	-2.032467	2.679425	-0.759	0.44868	
MFE_151	0.008537	0.006816	1.252	0.2113	
Pair-prob	0.061488	0.22621	0.272	0.78594	
Lag3	0.031727	0.05551	0.572	0.56802	
Lag2	0.179942	0.058943	3.053	0.00246	**
Lag1	0.371884	0.056404	6.593	1.76E-10	***
